# An early analysis of cost-utility of baroreflex activation therapy in advanced chronic heart failure in Germany

**DOI:** 10.1186/s12872-018-0898-x

**Published:** 2018-08-09

**Authors:** Oleg Borisenko, Jochen Müller-Ehmsen, JoAnn Lindenfeld, Erik Rafflenbeul, Christian Hamm

**Affiliations:** 1 Synergus AB, Danderyd (Stockholm), Sweden; 2 Department of Internal Medicine 3, Asklepios Klinik Altona, Hamburg, Germany; 3 Heart Failure and Transplantation Section Vanderbilt Heart and Vascular Institute Medical Center, Nashville, TN USA; 4 Department of Internal Medicine 1, Asklepios Klinik Barmbek, Hamburg, Germany; 5 Kerckhoff Heart and Thoraxcenter, Bad Nauheim and Medical Clinic I, University of Giessen, Giessen, Germany

**Keywords:** Baroreflex activation therapy, Barostim, Cost-utility, Health economics, Markov modeling, Germany

## Abstract

**Background:**

This study aimed to evaluate cost-utility of baroreflex activation therapy (BAT) using the Barostim *neo*™ device (CVRx Inc., Minneapolis, MN, USA) compared with optimized medical management in patients with advanced chronic heart failure (NYHA class III) who were not eligible for treatment with cardiac resynchronization therapy, from a statutory health insurance perspective in Germany over a lifetime horizon.

**Methods:**

A decision analytic model was developed using the combination of a decision tree and the Markov process. The model included transitions between New York Heart Association (NYHA) health states, each of which is associated with a risk of mortality, hospitalization, cost, and quality of life. The effectiveness of BAT was projected through relative risks for mortality (obtained by application of patient-level data to the Meta-analysis Global Group in Chronic Heart Failure risk prediction model) and hospitalization owing to worsening of heart failure (obtained from BAT Randomized Clinical Trial). All patients were in NYHA class III at baseline.

**Results:**

BAT led to an incremental cost of €33,185 (95% credible interval [CI] €24,561–38,637) and incremental benefits of 1.78 [95% CI 0.45–2.71] life-years and 1.19 [95% CI 0.30–1.81] quality-adjusted life-years (QALYs). This resulted in an incremental cost-effectiveness ratio of €27,951/QALY (95% CI €21,357–82,970). BAT had a 59% probability of being cost-effective at a willingness-to-pay threshold of €35,000/QALY (but 84% at a threshold of €52,000/QALY).

**Conclusions:**

BAT can be cost-effective in European settings in those not eligible for cardiac resynchronization therapy among patients with advanced heart failure.

## Background

Despite treatment advances over the past decade, heart failure (HF) remains a major burden on patients and healthcare systems. HF is the leading cause of hospitalization for patients who are older than 65 years, with an average length of stay of 13 days and a re-hospitalization rate of approximately 20% in the UK [1, 2]. In Europe, HF accounts for between 1.1 and 2.0% of the total health expenditure in healthcare [3].

The prevalence of HF in the general population is approximately 3.9% [4] and the annual incidence varies between 1.0 and 2.5 cases per 1000 population [5, 6]. The prognosis for HF patients remains generally poor. In the UK National Heart Failure Audit, the overall in-hospital and 1-year mortality of HF was 9.4 and 24.6%, respectively. In patients with reduced left ventricular ejection fraction (LVEF), 1-year mortality was approximately 15%, increased to 22–25% at 3 years, and reached 72% at 10 years [7, 8]. Patients with HF have a reduced quality of life and are often limited in their activities of daily living because of symptoms, such as dyspnea and fatigue. The quality of life of patients with HF is lower than that in those with angina pectoris, breast cancer, or diabetes mellitus [9]. The quality of life is reduced as symptoms worsen (New York Heart Association [NYHA] class) [10].

Current treatment options include pharmacotherapy and cardiac resynchronization therapy (CRT). However, CRT is not suitable for all patients [1]. In a UK study, only approximately 10% of patients presenting with HF were eligible for CRT [11]. Of those, between 25 and 35% of patients do not appear to respond to CRT [12].

Implantable cardioverter defibrillators only have a preventive effect on sudden cardiac death and do not improve symptoms or slow the progression of HF. Evidence of the beneficial use of implantable cardioverter defibrillators is less in patients with an LVEF of 30–35%, as well as in those with HF from a non-ischemic etiology [1, 13]. The use of ventricular assist devices and heart transplants is limited in European countries and they cannot be considered as a treatment option for the broad HF group of patients [14–16].

The Barostim *neo™* (CVRx Inc., Minneapolis, MN, USA) is a CE-marked treatment option for HF patients. This device is indicated for patients with heart failure NYHA class III with an LVEF ≤35%. The Barostim *neo*™ is an implantable medical device, which elicits the body’s natural baroreflex through stimulation of the carotid baroreceptors. This therapy is expected to restore the sympatho-vagal balance, which is a central physiological mechanism and therapeutic target in HF while preserving blood pressure and renal function. This therapy reduces the workload of the heart by decreasing arterial resistance, thereby improving the heart’s ability to pump blood to the tissues. The mechanism of action is not linked to dyssynchrony. Thus, stimulation of the baroreflex can help patients who do not have an indication for or have not fully responded to CRT. Therefore, the Barostim *neo*™ is both compatible with and complementary to CRT.

In a randomized controlled trial (*n* = 146; NCT01471860 and NCT01720160) versus guideline-directed medical therapy, BAT demonstrated improvements in the 6-min walk test (*p* = 0.004), quality-of-life score (*p* < 0.001), and NYHA class at 6 months (*p* = 0.002). BAT was also associated with a trend towards a reduction in days of hospitalization due to HF (*p* = 0.08) [17]. In a follow-up publication by Zile et al. [18], the clinical effect of BAT was more profound in patients without CRT. There was a significant reduction in the number of hospitalizations (reduction by 0.53 ± 0.2, *p* < 0.05) and days of hospitalization due to HF (reduction by 8.89 ± 4.0 days, *p* < 0.05) in the BAT arm compared with the 6 months prior to enrollment, whereas no difference was found in the control arm. However, a reduction in resource use at 6 months compared with the control arm was marginal (*p* = 0.08 for hospitalizations and *p* = 0.09 for days of hospitalization). Despite promising clinical data, economic consequences of the use of BAT have not been evaluated.

Therefore, the objective of this study was to determine the cost utility of BAT as a treatment in adult patients with advanced HF and reduced ejection fraction (defined as LVEF < 35% and NYHA class III) compared with the relevant pharmacological multi-drug treatment in a German population from a healthcare payer perspective over a lifetime horizon. The study focused on the population of patients without CRT, with the most profound clinical effects of the BAT.

## Methods

### Model structure

**Fig. 1:**
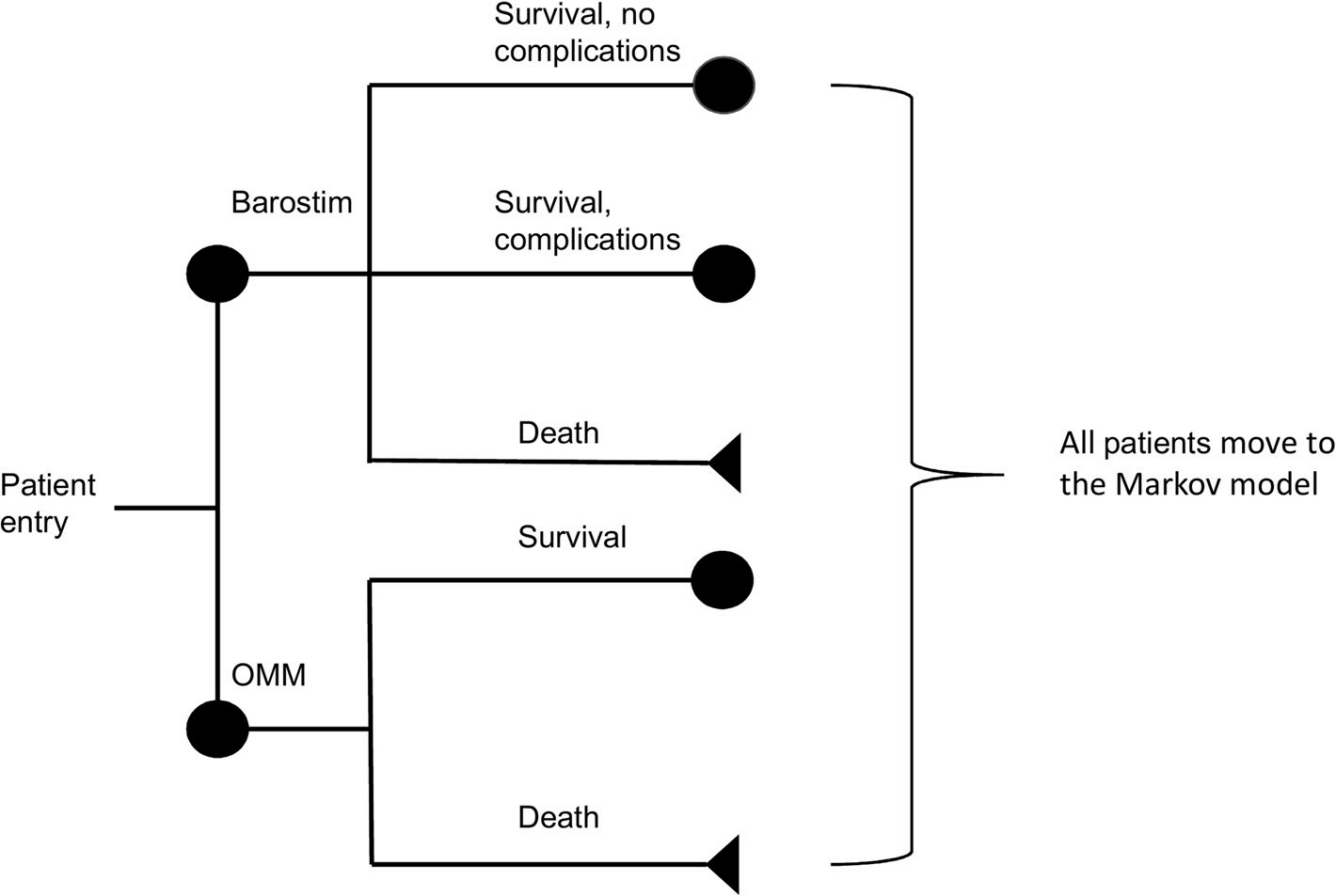
Structure of decision tree

A combination of a decision tree and Markov modeling was used. The short-term model (a decision tree) covers the time span of the first 30 days after receiving implantation for the BAT arm or receiving purely optimized medical management (OMT). The structure of the decision tree is shown in Fig. 1. Patients in the BAT arm could survive without complications, survive with complications, or die during the procedure. Patients in the OMT arm could either survive or die.

**Fig. 2:**
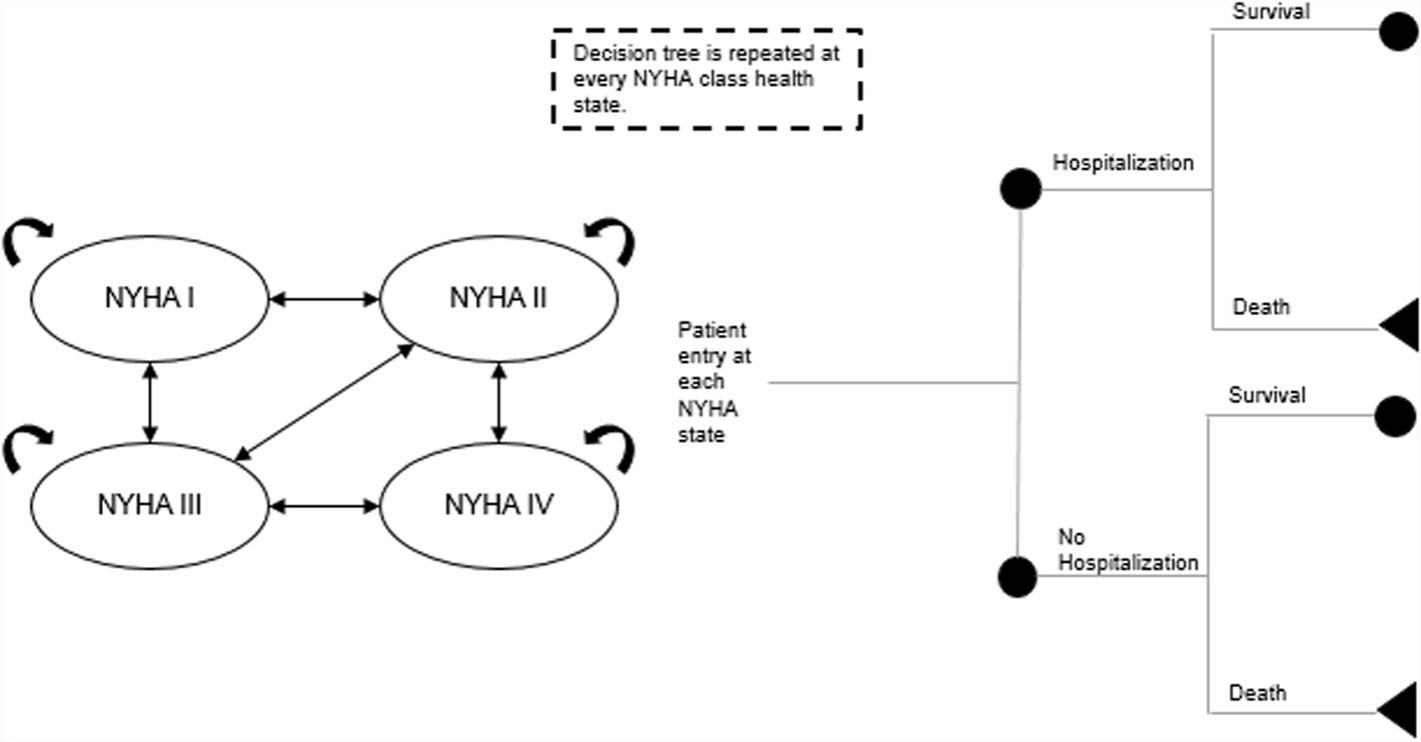
Structure of Markov model. NYHA, New York Heart Association. Figure is reproduced with permission from Borisenko O, Haude M, Hoppe UC, Siminiak T, Lipiecki J, Goldberg SL, et al. Cost-utility analysis of percutaneous mitral valve repair in inoperable patients with functional mitral regurgitation in German settings. BMC Cardiovasc Disord. 2015;15:43. doi: 10.1186/s12872-015-0039-8

The structure of the Markov model was adopted from the decision analytic model of Ford et al. [19] (Fig. 2). The Markov model follows the natural history of the disease and is based on five health states: NYHA functional classes I, II, III, and IV and death. In all health states, patients can remain in the same state, have improvement or deterioration of symptoms, and have a probability of dying because of heart failure. Within each state, patients have a probability of being hospitalized because of worsening of symptoms of heart failure. Hospitalized and non-hospitalized patients have the same probability of dying. Cycle length is 1 month. This means that patients can transit between different health states only once per month. Patients start in the Markov model from the first cycle.

### Data input

#### Clinical and safety data

Basic probabilities of patient’s progression between NYHA classes for the first cycle, second cycle, and onwards were the same for both arms (however, they were adjusted for the BAT arm, using the approach described below) and were obtained from the CARE-HF trial [20]. The probability of hospitalization and excess mortality (on top of normal mortality) by NYHA class was obtained from the literature [19] (Table 1). No excess risk of death was assumed for NYHA class I. Mortality in the overall population was obtained from German sex-specific life tables.

**Table 1:** Probability of hospitalization, excess mortality for hospitalized states and probability of adverse events

Parameter	Value	Variance	Distribution and coefficients for probabilistic sensitivity analysis	Source
Six-month probability of excess mortality for NYHA class II	0.04	0.032–0.048	Beta (α = 4; β = 96)	Cowper 2004 [35]
Six-month probability of excess mortality for NYHA class III	0.07	0.056–0.084	Beta (α = 7; β = 93)	
Six-month probability of excess mortality for NYHA class IV	0.28	0.224–0.336	Beta (α = 28; β = 72)	
Monthly probability of hospitalization for NYHA class I	0.015	0.008–0.023	Beta (α = 1.5; β = 98.5)	Ford 2012 [19]
Monthly probability of hospitalization for NYHA class II	0.024	0.012–0.036	Beta (α = 2.4; β = 97.6)	
Monthly probability of hospitalization for NYHA class III	0.024	0.012–0.036	Beta (α = 2.4; β = 97.6)	
Monthly probability of hospitalization for NYHA class IV	0.154	0.77–0.23	Beta (α = 15.4; β = 84.6)	
Probability of short-term adverse events (30 days)	0.070	0.03–0.1	Beta (α = 3; β = 38)	Barostim Clinical evidence report (unpublished)
Probability of serious adverse event (Months 1 to 6)	0.033	0–0.05	Beta (α = 1; β = 29)	Hoppe 2012 [36]
RR for mortality in Barostim arm	0.61	0.52–0.70	Log-normal (SE_log_ = 0.0467)	Application of the data from the MAGGIC risk prediction model to the individual patient data from the RCT of Barostim in heart failure
RR for hospitalizations due to heart failure in Barostim arm	0.40	0.34–1.05	Log-normal (SE_log_ = 0.18)	Zile 2015 [18]

The effectiveness of BAT was modeled via application of relative risks for all-cause mortality and hospitalizations compared with OMT. These relative risks were assumed to be independent of NYHA class. The relative risk for mortality was obtained from the application of anonymized individual patient-level data from the BAT Randomized Controlled Trial [17] to the Meta-analysis Global Group in Chronic Heart Failure (MAGGIC) risk prediction model [21]. The MAGGIC model provides a comprehensive opportunity to develop a prognostic model in patients with HF, with reduced and preserved LVEF. This model uses readily available risk factors based on 39,372 patients with HF from 30 cohort studies, six of which were randomized controlled clinical trials (24,041 patients) and 24 were registries (15,331 patients). A final model included 13 highly significant independent predictors of mortality in the following order of predictive strength: age, lower ejection fraction, NYHA class, serum creatinine, diabetes, no prescription of a beta-blocker, lower systolic blood pressure, lower body mass, time since diagnosis, current smoker, chronic obstructive pulmonary disease, male sex, and no prescription of an angiotensin-converting enzyme inhibitor or angiotensin-receptor blocker. Although these independent predictors of mortality in HF have been previously identified, the MAGGIC model and risk score are the most comprehensive and generalizable items available in the literature. Analysis of 1-year risk of mortality was performed in 82 patients (who had available LVEF and NYHA class information at 6-month follow-up), 52 patients were in the device arm, and 30 patients in the control arm. For each patient, an individual total integer risk score was calculated. From this score, a predicted probability of death was estimated. Three variables were not available in the BAT Randomized Controlled Trial (smoking status, the presence of chronic obstructive pulmonary disease, and first diagnosis of HF in the previous 18 months) and they were obtained from the original cohort in the MAGGIC meta-analysis [21]. For these variables, a list of values was developed following appropriate distribution and values from the list were randomly assigned to each patient in the cohort.

The relative risk of mortality at 1 year was 0.61. The relative risk for hospitalization (relative risk = 0.40) was obtained from a study by Zile et al. [18] (16% were hospitalized in the BAT arm and 40% in control arm). Relative risks were assumed to be constant and were applied over a lifetime horizon.

Data on the rate of serious complications and mortality following implantation of BAT were obtained from a combined experience with the Barostim *neo*™ device in HF and resistant hypertension. Short-term (30-day) complications included intraoperative bleeding with transfusion, edema to surgical incision, and pain and tingling around the surgical wound. Six-month complications included intermittent pain near the device system.

#### Cost inputs

All cost inputs were based on German data (Table 2). The cost of the BAT implant procedure (G-DRG 901D, German Diagnosis Related Groups) and the battery replacement (G-DRG F17B) procedures were derived from the German DRG in 2013. To estimate the cost of the procedure (except for the cost of the device), the length of hospital stay was assumed to be 2 days, and the cost of the implant was subtracted from the overall DRG. The remaining value was summed up with the cost of the full Barostim *neo*™ system or the cost of the battery for a replacement procedure.

**Table 2:** Cost inputs

Variable	Value	Variance	Distribution and coefficients for probabilistic sensitivity analysis	Source
Cost of Barostim implant procedure, €	3628	1814–5442	–	G-DRG 901D 2013, LOS = 2 days, cost of implant was subtracted
Cost of battery replacement procedure, €	1808	904–2712	–	G-DRG F17B 2013, LOS = 2 days, cost of implant was subtracted
Cost of pre- and post-implant physician visits, €	68	34–102	–	EBM 13542 (1 visit), EBM 07212 (2 visits)
Cost of full Barostim system, €	21,000	15,000–24,000	–	CVRx Inc.
Cost of Barostim battery, €	15,000	10,000–17,000	–	CVRx Inc.
Cost of short-term complications, €	3056	1528–4584	–	G-DRG 901D 2013. The difference between 2 and 5-day LOS. It was assumed that complication will lead to prolongation of hospital stay
Cost of long-term complications, €	0	0–100	–	Assumption
Battery life, years	6	3–6	–	CVRx Inc.
Percentage of patients being hospitalized with stay in intensive care unit	7.2%	–	–	Yao 2007 [20]
Percentage of patients being hospitalized with stay in coronary care unit	25.6%	–	–	
Percentage of patients being hospitalized with CABG performed	0.3%	–	–	
Percentage of patients being hospitalized with PTCA performed	0.2%	–	–	
Percentage of patients being hospitalized with heart transplantation performed	2.6%	–	–	
Percentage of patients being hospitalized with no procedure performed	62.3%	–	–	
Cost of hospitalization with stay in intensive care unit, €	5005	2502–7507	–	G-DRG code F62A 2013
Cost of hospitalization with stay in coronary care unit, €	5004	2502–7507	–	G-DRG code F62A 2013
Cost of hospitalization with CABG performed, €	15,056	7528–22,584	–	G-DRG code F06E
Cost of hospitalization with PTCA performed, €	3793	1896–5689	–	G-DRG code F56B plus ZE101
Cost of hospitalization with heart transplantation performed, €	86,337	43,169–129,507	–	G-DRG code A05B 2013
Cost of hospitalization with no procedure performed, €	2740	1370–4110	–	G-DRG code F62B 2013
Yearly cost of routine management NYHA I, €	516	258–1031	Gamma (α = 1;λ = 516)	Biermann 2012 [22]
Yearly cost of routine management NYHA II, €	910	455–1821	Gamma (α = 1;λ = 910)	
Yearly cost of routine management NYHA III, €	900	450–1800	Gamma (α = 1;λ = 900)	
Yearly cost of routine management NYHA IV, €	967	484–1935	Gamma (α = 1;λ = 967)	

The cost of short-term complications was assumed as the difference between 2 and 5 days of hospital stay, where it was assumed that complications will lead to a prolonged hospital stay. The cost of pre- and post-implant physicians’ visits were derived from the out-patient statutory health insurance reimbursement catalog (Einheitlicher Bewertungsmaβstab, EBM) using codes 13,542 and 07212. We assumed that one pre-implant and two post-implant visits will be required. We also assumed that long-term complications (pain near stimulator) do not lead to consumption of healthcare resources and do not have any cost.

The cost of the full Barostim *neo*™ system and battery were provided by CVRx Inc. The battery life was 6 years.

The cost of routine management of HF (including costs of visits to physician, medication, and rehabilitation, but excluding costs of hospitalization owing to worsening of HF) according to different NYHA classes was obtained from the literature [22]. The costs of hospitalization with intensive care units, coronary care units, coronary artery bypass grafting, percutaneous transluminal coronary angioplasty, transplantation, and no interventional procedures performed were obtained from the German DRGs. The distribution of reasons for hospitalizations was obtained from the literature [20]. All cost data are presented in 2013 Euros. Inflation adjustment was performed using the German consumer price index [23].

#### Utility inputs

Utility scores were assigned for each NYHA class irrespective of received treatment and hospitalization status. Utility values and corresponding distribution parameters were obtained from the CARE-HF trial, in which they were measured using the EQ-5D [19, 20]. The lower and upper bounds of the range for the one-way deterministic sensitivity analysis were obtained by reducing and increasing the baseline values of the utilities by 20%. Utility weights were combined with life-years to estimate QALYs.

#### Statistical analysis

The target population included patients who were not eligible for CRT according to established guidelines. The patients had a mean age of 63 years, 83.2% were male, and had chronic heart failure in NYHA class III.

The incremental cost-effectiveness ratio (ICER) was calculated by comparing the difference in average total costs with the difference in average quality-adjusted life years (QALYs) between two patient cohorts. The intervention was considered cost-effective if the ICER was below €35,000 per QALY. All costs and outcomes beyond the first year were discounted 3.0% annually based on the recommendations of the German National Institute for Quality and Efficiency in Health Care (IQWiG) [24]. The model was constructed using Microsoft Excel 2013 (Microsoft Corp., Redmond, WA, USA).

In the base-case analysis results were obtained from probabilistic sampling of the model with 5000 simulations. Unlike in probabilistic sensitivity analysis, only patient’s gender and age were sampled, while keeping all other inputs (costs, transition probabilities, utilities) fixed. Mean values and 95% credible intervals were reported for costs, QALY and LYG. A normal distribution was assigned to patient age, whereas patient gender was assumed to follow a beta distribution.

#### Sensitivity analysis

One-way sensitivity analysis was performed to assess the effect of varying model parameters while holding other variables fixed at base-case values. Cost drivers (variables with major effect on ICER) were identified and results were presented using a Tornado diagram. In addition, a probabilistic sensitivity analysis was performed using Monte Carlo simulations. Five thousand simulations were performed. Beta distribution was used for the probabilities and utilities. Gamma distribution was used for cost data. Log-normal distribution was used for the relative risks. Dirichlet distribution was used for the probabilities of transition between NYHA classes.

#### Model validation

The performance of the model was tested against data from within-trial, all-cause mortality outcomes in the medical treatment group of the CARE-HF trial [25]. To perform validation, the baseline patients’ characteristics in the model were adjusted to mean (standard deviation) age (66 ± 6.6 years) and the proportion of male individuals (73%) of patients in the CARE-HF trial, and 5000 Monte Carlo simulations were performed.

## Results

### Model validation

Validation showed that the model precisely predicted all-cause mortality, with results of the modeling (all-cause mortality at 30 months) estimated as 31% vs 30% in the trial.

### Base-case results

In the analysis, BAT led to an incremental cost of €33,185 (95% credible interval €24,561–38,637) and incremental benefits of 1.78 (95% credible interval 0.45–2.71) life-years and 1.19 (95% credible interval 0.30–1.81) QALYs (Table 3). This resulted in an ICER of €27,951/QALY (95% credible interval €21,357–82,970).

**Table 3:** Results of cost-utility analysis

	Cost, €	∆ cost €	Life years gained	∆ LYG	QALYs gained	∆ QALY	ICER, €/QALY
BAT	50,856 [34,358-59,437]	33,185 [24,561-38,637]	7.25 [3.44–9.17]	1.78 [0.45–2.71]	4.86 [2.30–6.14]	1.19 [0.30–1.81]	27,951 [21,357-82,970]
OMM	17,671 [9796-20,800]		5.47 [3.00–6.46]		3.67 [2.00–4.33]		

### Sensitivity analysis

**Fig. 3:**
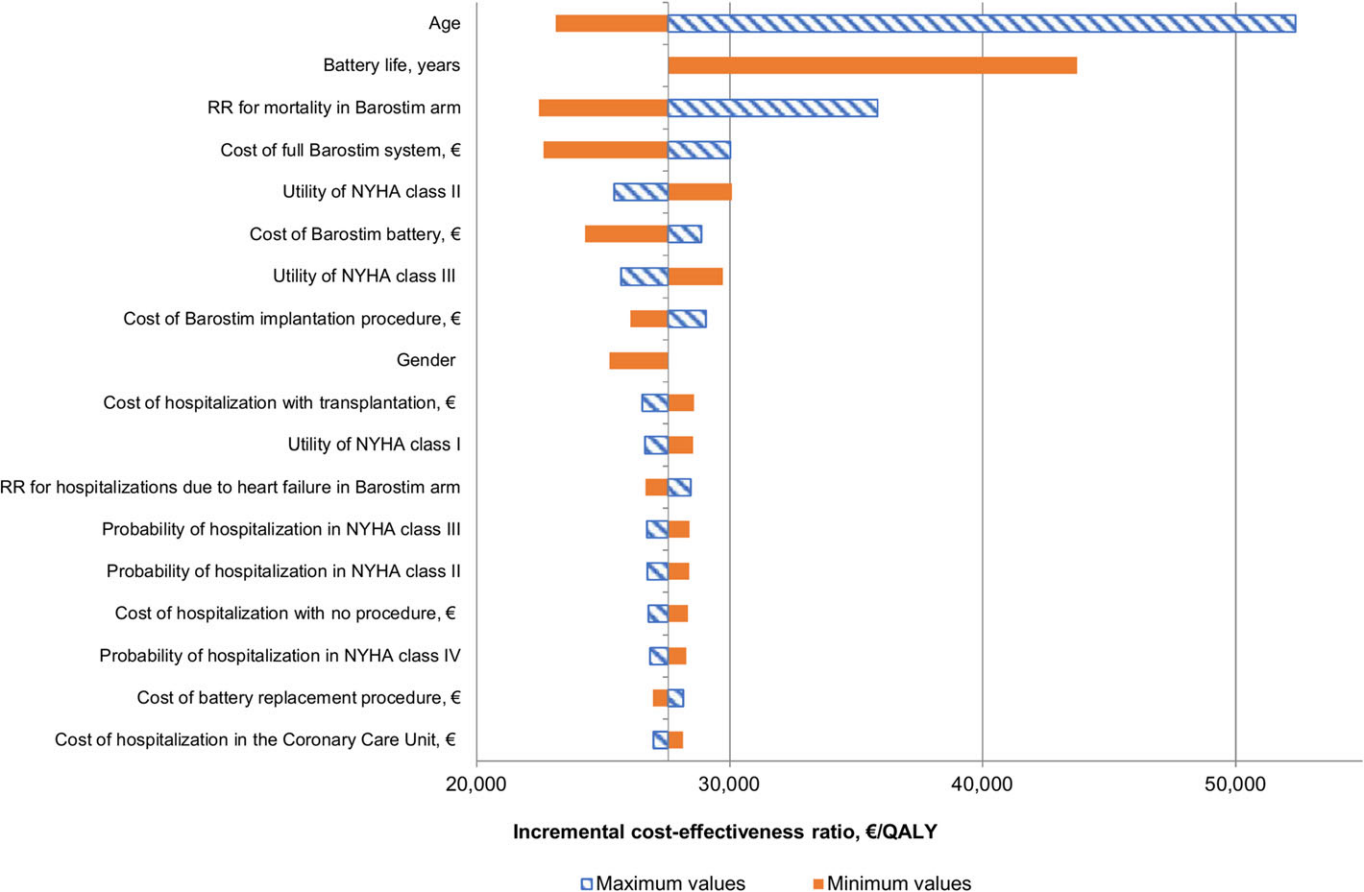
Tornado diagram

**Fig. 4:**
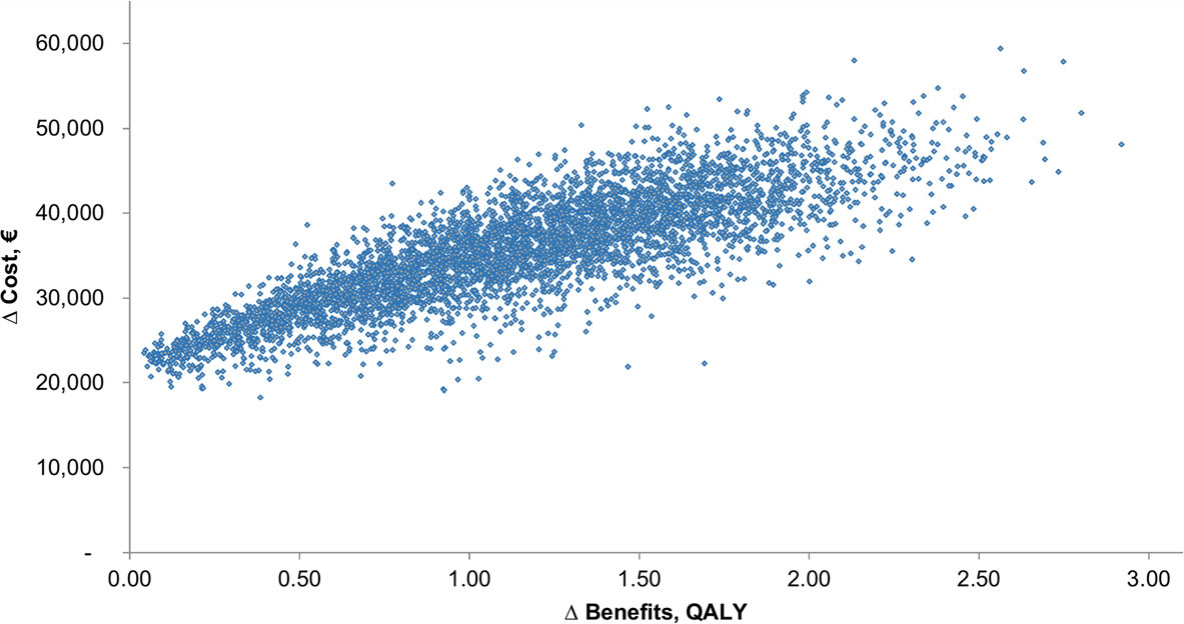
Cost-effectiveness acceptability plane

**Fig. 5:**
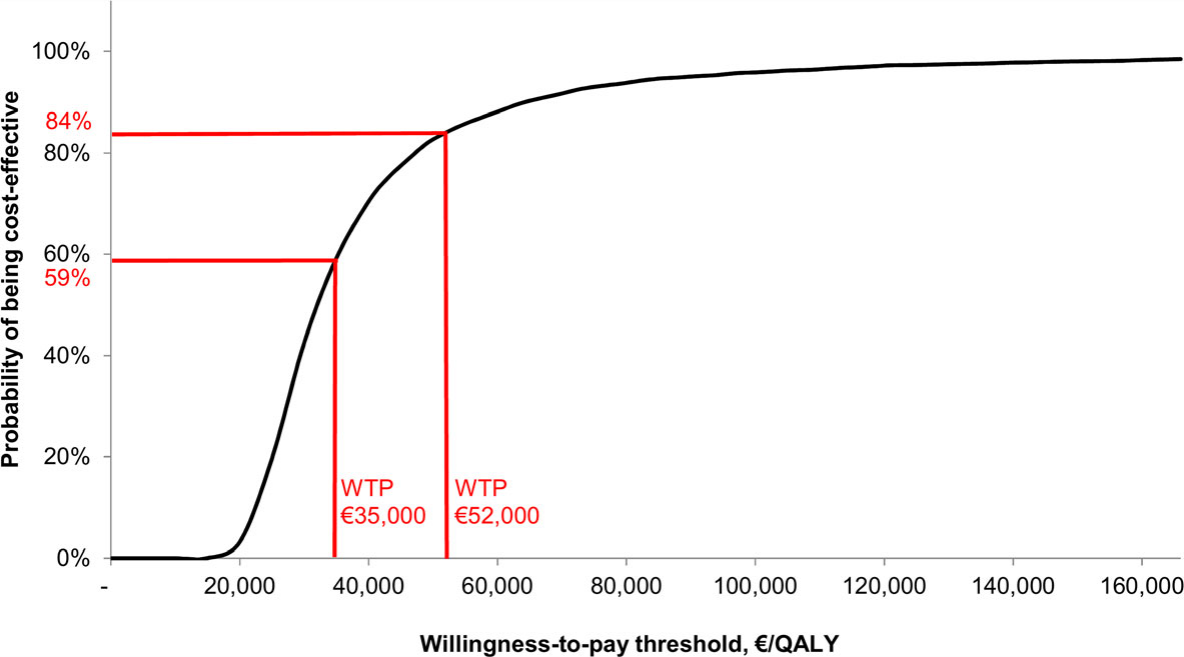
Cost-effectiveness acceptability curve. WTP, willingness-to-pay threshold

Deterministic one-way sensitivity analysis showed that the most sensitive parameters were age (BAT is more cost-effective in younger patients), battery life (a shorter battery life reduced the cost-effectiveness of BAT), and relative risk for mortality (a higher relative risk reduces the cost-effectiveness of BAT) (Fig. 3). Probabilistic sensitivity analysis demonstrated that BAT led to an increase in the cost of treatment and additional benefits in the majority of simulations (Fig. 4). At a willingness-to-pay threshold of €35,000/QALY, BAT had a 59% probability of being cost-effective but reached an 84% probability of being cost-effective at a threshold of €52,000/QALY (Fig. 5).

## Discussion

Our study evaluated potential long-term clinical and economic consequences of BAT compared with optimal medical treatment over a lifetime in a cohort of patients aged 63 years with NYHA class III at the start of the treatment. We showed that BAT leads to additional costs to a health care system (on average €33,185). However, BAT also could lead to additional benefits in terms of survival (an average additional 1.78 life-years) or quality-adjusted survival (an average additional 1.19 QALYs). These results indicate that BAT can be cost-effective because the resulting ICER is below the typical willingness-to-pay threshold in European countries (€35,000/QALY). In the probabilistic sensitivity analysis, the most preferred analysis to simultaneously address all data-related uncertainty in the model, BAT had an approximately 60% chance of being cost-effective at a willingness-to-pay threshold of €35,000/QALY. The potential correlation between input parameters was not captured, which might be associated with a limitation in interpreting the results of the PSA.

However, these promising findings need to be evaluated in the context of existing limitations of our analysis. First, data on the effect of BAT on mortality were obtained via application of a risk prediction model to individual patient-level data. They were not derived directly from the randomized controlled trial because these data are not yet available. Risk prediction models are used in the field of epidemiology to provide estimations of the absolute probabilities of the occurrence of a certain outcome in an individual with a specific set of characteristics or predictors. Predictors may vary from age and sex to advanced diagnostic markers, such as natriuretic peptides or the genetic profile [26]. Models are usually developed from large datasets using multivariable regression modeling [27]. The ultimate aim of a model is to assist clinicians and patients in decision making regarding the further management of HF, as well as to facilitate timely research [28, 29]. To be applied correctly, risk prediction models should provide accurate and validated probability estimates for the outcomes of interest in a targeted population. This can be ensured by the internal and external validation of the model by means of statistical methods [26].

There are several other risk scores models for predicting survival for patients with HF. These include the Seattle heart failure model [30], the Heart Failure Survival Score [31], the PACE Risk Score [32], and SHOCKED Predictors [33]. A systematic literature review identified 20 different risk prediction models [34]. Each model uses a single cohort of patients and thus has more limited generalizability to other populations. Additionally, each model’s development is from a limited cohort size, compromising the ability to truly quantify the best risk prediction model. Because of the wide variety of different studies included, with a global representation, the findings in the MAGGIC meta-analysis appear to be inherently generalizable to a broad spectrum of current and future patients. The risk score, developed in the MAGGIC meta-analysis of a large dataset of 30 cohort studies, provides a uniquely robust and generalizable tool to quantify individual patients’ prognosis in HF.

A second limitation is that data on the effect of BAT on the rate of hospitalizations were derived from the BAT Randomized Controlled Trial, in which a trend towards a between-group difference in the number of hospitalizations was observed, but this was not statistically significant [18]. The original study did not have sufficient power to demonstrate statistical significance in this outcome in a subgroup of patients with no CRT. This outcome shall be evaluated in a further study of BAT. However, the use of the data is justifiable in our exploratory analysis, focusing on understanding the potential of BAT as a treatment option for advanced HF. Moreover, in the sensitivity analysis, an increase in relative risk to 1.05 (indicating that BAT will lead to an increased risk of hospitalizations) only led to a marginal increase in the ICER over the willingness-to-pay threshold (ICER was €37,172/QALY).

## Conclusion

In conclusion, results of the preliminary cost-utility analysis indicate that baroreflex activation therapy can be cost-effective in European settings at the commonly accepted willingness-to-pay threshold of €35,000/QALY in patients with advanced HF who are not eligible for CRT.

## Abbreviations

BAT: Baroreflex activation therapy
CI: Credible interval
CRT: Cardiac resynchronization therapy
G-DRG: German Diagnosis Related Groups
HF: Heart failure
ICER: Incremental cost-effectiveness ratio
LVEF: Left ventricular ejection fraction
LYG: Life-year gained
MAGGIC: Meta-analysis Global Group in Chronic Heart Failure
NYHA: New York Heart Association
OMT: Optimized medical management
PSA: Probabilistic sensitivity analysis
QALY: Quality-adjusted life-year
WTP: Willingness-to-pay threshold
